# F18-choline PET/CT guided surgery in primary hyperparathyroidism when ultrasound and MIBI SPECT/CT are negative or inconclusive: the APACH1 study

**DOI:** 10.1007/s00259-017-3911-1

**Published:** 2017-12-22

**Authors:** Elske Quak, David Blanchard, Benjamin Houdu, Yannick Le Roux, Renaud Ciappuccini, Barbara Lireux, Dominique de Raucourt, Jean-Michel Grellard, Idlir Licaj, Stéphane Bardet, Yves Reznik, Bénédicte Clarisse, Nicolas Aide

**Affiliations:** 1 Department of Nuclear Medicine and Thyroid Unit, François Baclesse Cancer Centre, Caen, France; 2 Department of Head & Neck Surgery, François Baclesse Cancer Centre, Caen, France; 3 Department of Nuclear Medicine, University Hospital, Caen, France; 4 Department of General and Endocrine Surgery, University Hospital, Caen, France; 5 INSERM 1086 ANTICIPE, Caen University, Caen, France; 6 Department of Clinical Research, François Baclesse Cancer Centre, Caen, France; 7 Department of Endocrinology, University Hospital, Caen, France

**Keywords:** Parathyroid adenoma, Primary hyperparathyroidism, MIBI SPECT/CT, F18-choline, PET/CT, Minimally invasive surgery

## Abstract

**Purpose:**

To evaluate the sensitivity of F18-choline (FCH) PET/CT for parathyroid adenoma detection prior to surgery in patients with primary hyperparathyroidism and negative or inconclusive cervical ultrasound and Tc99m-sestaMIBI SPECT/CT.

**Methods:**

We conducted a prospective bicentric study (NCT02432599). All patients underwent FCH PET/CT. The result was scored positive, inconclusive or negative. The number of uptakes and their sites were recorded. The FCH PET/CT result guided the surgical procedure (minimally invasive parathyroidectomy, bilateral cervical exploration, or other in case of multiple or ectopic foci). FCH PET/CT results were compared to the surgical and pathological findings and the follow-up.

**Results:**

Twenty-five patients were included. Mean calcium and PTH levels prior to surgery were 2.76 ± 0.17 mmol/l and 94.8 ± 37.4 ng/l. Nineteen (76%) FCH PET/CTs were scored positive, 3 (12%) inconclusive and 3 (12%) negative, showing 21 cases of uniglandular disease, including 1 ectopic localization and 1 case of multiglandular (3 foci) disease. Mean lesion size was 13.1 ± 8.6 mm. Twenty-four patients underwent surgery. FCH PET/CT guided surgery in 22 (88%) patients, allowing for 17 minimally invasive parathyroidectomies, 1 bilateral cervical exploration for multifocality and 4 other surgical procedures. Two patients with negative FCH-PET/CT underwent bilateral cervical exploration. When dichotomizing the FCH PET/CT results, thereby classifying the inconclusive FCH PET/CT results as positive, the per lesion and per patient sensitivities were 91.3% (95%CI: 72.0–98.9) and 90.5% (95%CI: 69.6–98.8) and the corresponding positive predictive values were 87.5% (95%CI: 67.6–97.3) and 86.4% (95%CI: 65.1–97.1), respectively.

Twenty-one (88%) patients were considered cured after surgery. Their mean calcium level after surgery was 2.36 ± 0.17 mmol/l.

**Conclusions:**

Preoperative FCH PET/CT has a high sensitivity and positive predictive value for parathyroid adenoma detection in patients with primary hyperparathyroidism and negative or inconclusive conventional imaging results. Bilateral cervical exploration could be avoided in the majority (75%) of patients.

## Introduction

Preoperative localisation of hyperfunctioning parathyroid tissue in primary hyperparathyroidism (PHPT) is a prerequisite for minimally invasive parathyroidectomy (MIP). Currently, the most frequently used imaging methods are cervical ultrasound and Tc99m-sestamibi (MIBI) parathyroid scintigraphy. Cervical ultrasound is a non-invasive, non-irradiating, low-cost and readily available imaging modality for parathyroid imaging, and it allows for the analysis of any concomitant thyroid nodules [1]. However, its detection rate and sensitivity for parathyroid adenoma (PTA) imaging are mediocre; reported sensitivities vary from 57% to 76% [2–4], and deep-laying or ectopic PTAs will go undetected [4, 5]. Parathyroid scintigraphy, ideally including a SPECT/CT acquisition, is a non-invasive, slightly irradiating and readily available imaging method allowing for the detection of approximately two-thirds of PTAs in our population [6]. Reported sensitivities of parathyroid scintigraphy including SPECT/CT, mainly from retrospective studies, vary from 53% to 92% [3, 6–8]. In contrast to ultrasound, scintigraphy can detect deep-laying or ectopic PTAs.

In case of positive imaging for a single focus, a MIP can be performed, often on an outpatient basis and sometimes under local anesthesia. This procedure is known to be superior in terms of cure and complication rates compared to the conventional inpatient bilateral cervical exploration (BCE) performed in case of negative imaging procedures, and is less costly [9].

Recently, collaborators in this trial coincidentally detected a PTA with F18-choline (FCH) PET/CT in a male patient referred for biochemical recurrence of prostate cancer [10]. Since, several small studies have shown promising results for the detection of hyperfunctioning parathyroid tissue with FCH PET/CT compared to ultrasound and parathyroid scintigraphy [11–13]. Potential advantages of FCH PET over parathyroid scintigraphy are an improved spatial resolution allowing for detection of smaller lesions, and a shorter study protocol due to the rapid biokinetics of choline [14, 15].

Therefore, we performed a prospective pilot study evaluating the sensitivity of FCH PET/CT as a second line nuclear medicine imaging technique for preoperative PTA detection in patients with PHPT and a negative or inconclusive ultrasound and MIBI SPECT/CT. Secondary aims of this study were i) to evaluate the positive predictive value of FCH PET/CT for PTA detection, ii) to determine the optimal acquisition protocol for FCH PET/CT, iii) to estimate the number of avoided BCEs.

## Materials and methods

### Study population

Patients with a biologically proven PHPT (hypercalcemia and elevated or inappropriately normal parathyroid hormone (PTH) levels) and negative or inconclusive ultrasound and MIBI SPECT/CT results prior to surgery were prospectively included. Exclusion criteria were severe kidney failure (creatinine clearance <30 ml/min), profound Vitamin D deficiency or the Multiple Endocrine Neoplasia-1 (MEN-1) syndrome. The local ethics committee approved the study protocol (Ref. 2014–41, Comité de protection des personnes Nord-Ouest III). All patients gave written informed consent. This trial is registered as EUDRACT 2014–003852-30, Clinical trial NCT02432599.

### MIBI SPECT/CT and ultrasound

All patients underwent cervical ultrasound and MIBI SPECT/CT prior to inclusion. Ultrasound was performed by local or external radiologists. Results were classified as negative or inconclusive according to the written report. The presence or absence of concomitant thyroid nodules was recorded.

MIBI SPECT/CT was performed identically in the two participating centers on SymbiaT2 systems (Siemens Medical Solutions). After intravenous injection of 740 MBq of MIBI, an early pinhole acquisition of the anterior lower neck was performed 10 min post-injection, followed by a late SPECT/CT acquisition of the neck and upper chest 90 min post-injection. A detailed description of the MIBI SPECT/CT protocol can be found elsewhere [6].

Images were interpreted by an experienced nuclear medicine physician (EQ, NA). A negative MIBI SPECT/CT was defined as the absence of focal uptake on the early and delayed images. An inconclusive MIBI SPECT/CT result was defined as faint uptake compared to the surrounding background without CT substrate or uptake most likely related to a thyroid nodule.

### Biology

Upon inclusion, serum values of calcium, parathyroid hormone (PTH), albumin, phosphorus, vitamin D and creatinine were measured for all patients, and the creatinine clearance was calculated according to the MDRD formula [16].

### FCH PET/CT

Sixty minutes after intravenous injection of 1,5 MBq/kg of FCH, a low-dose CT was performed (CAREdose ref. mAs 100, 130 kV, slice 3 mm, pitch 1.0), followed by a one bed position PET acquisition of 10 min covering the neck and upper chest in 3D list-mode on a Biograph 6 TrueV PET/CT system (Siemens Medical Solutions). The injected activity and the exact delay between injection and the start of the acquisition were recorded. The effective dose due to the low-dose CT was calculated by multiplying the dose-length product with the conversion factor 0.0059 mSv/mGy/cm [17], and the effective dose due to the FCH administration by multiplying the injected dose in MBq by the conversion factor 0.019 mSv/MBq [18]. The raw PET data were iteratively reconstructed with 3 iterations, 21 subsets, point-spread-function (PSF) modeling (TrueX), matrix size 256*256 and zoom 1.0. The list mode data were reconstructed with an increment of 2 min (2 min, 4 min, 6 min, 8 min and 10 min). Scatter and attenuation corrections were applied. No post-reconstruction filter was used. Image analysis was performed on Leonardo workstations (Siemens Medical Solutions). Only the 10 min reconstruction was used for the clinical FCH PET/CT report.

The FCH PET/CT was considered positive in the case of clear focal uptake(s) in a predisposing area. The exact location of each focus was noted (the side and upper or lower position, or the ectopic position), and when measurable the maximum transverse CT diameter. The FCH PET/CT was considered inconclusive in the presence of faint focal uptake superior to the surrounding background without CT substrate and negative in the absence of focal uptake. A second blind read of all exams was performed 9 months after the last inclusion by the same readers in order to estimate the inter observer agreement.

The semi-quantitative analysis was performed with MIM-software (version 5.6, MIM Software Inc., Cleveland, OH). The PTA SUVmax measurement was performed by placing a VOI on the parathyroid uptake with the PET Edge tool. Background SUVmean was measured by placing a spherical VOI with a 1 cm diameter on the contralateral thyroid lobe, if present, and the contralateral sternocleidomastoid (SCM) muscle. The PTA-to-thyroid and PTA-to-SCM ratios were calculated for all (2 min, 4 min, 6 min, 8 min and 10 min) attenuation corrected reconstructions.

### Surgery

All patients underwent surgery within four weeks following the FCH PET/CT by a dedicated head and neck or endocrine surgeon in one of the participating centers. The surgeon had access to the clinical FCH PET/CT report. In case of a positive FCH PET/CT, an outpatient MIP was performed. The surgical procedure was adapted in the case of suspected multiple or ectopic PTAs. In case of an inconclusive FCH PET/CT, surgery was performed on the site of the dubious focus. In case of a negative FCH PET/CT, a conventional inpatient BCE was performed.

The exact location was noted for each resected specimen, as were the total surgery time and surgical complications if any.

### Histology

During surgery, an intra-operative frozen section was performed to confirm the presence of parathyroid tissue. Final analysis was performed on paraffin-wax embedded sections stained with hematoxylin and eosin. When necessary, immunohistochemistry with anti-PTH antibody was performed. Parathyroid adenoma and parathyroid hyperplasia were considered true positive.

### Outcome

In the days following surgery, the serum calcium level was repeated. Patients were considered cured in case of histological proof of PTA or parathyroid hyperplasia and a normalization of the serum calcium level after surgery.

### Statistical analysis

The planned sample size for the present single-stage design was based on the estimated sensitivity of FCH PET/CT for PTA detection [12] that should be superior to 0.60 to be sufficient. With unilateral *alpha* of 0.10, an anticipating sensitivity of 0.90 and a power = 0.80 we determined that 20 patients should be included to detect 12 PTAs. The sample size was increased to 24 to correct for drop-out. Quantitative variables were described with mean and standard deviations, whereas qualitative variables were described with numbers and percentages. The sensitivity and positive predictive value were calculated on a lesion and patient level respectively. The Wilcoxon rank-sum test was used for continuous variables. The kappa statistic according to Fleiss-Cuzick was used to determine the inter-observer agreement, with 95% confidence intervals using an inverted modified Wald test approach, as recommended by Zou and Donner [19]. Kappa values were interpreted as follows: <0 poor agreement, 0.0–0.20 slight agreement, 0.21–0.40 fair agreement, 0.41–0.60 moderate agreement, 0.61–0.80 substantial agreement, 0.81–1.00 almost perfect agreement. The nonparametric Friedman test was used to compare the ratios between SUV_max_ of the adenomas to the SUV_mean_ in background (either muscle or thyroid) amongst the different reconstructions. A post hoc test was performed with the Dunn test for multiple comparisons. For all tests, a two-tailed *P* value of 0.05 or less was considered statistically significant.

Analyses were performed with STATA, version 12 software (Stata Corp, College Station, TX) and Prism (GraphPad Software, La Jolla, CA).

## Results

### Patient characteristics

From March 2015 through February 2017, 25 patients were included and 24 patients completed the study protocol. The patient characteristics, blood test results and ultrasound and MIBI SPECT/CT results can be found in Table 1.

**Table 1:** Patient characteristics

Characteristic (*n* = 25)		
Sex ratio male/female	0.4	
Age (years), mean (SD)	58.9 (14.2)	
BMI (kg/m^2^), mean (SD)	28.4 (8.8)	
Previous thyroid surgery, n (%)	2 (8)	
Previous parathyroid surgery, n (%)	2 (8)	
Biology (serum levels), mean (±SD) (normal ranges)		
Calcium (mmol/l)	2.76 (0.17)	(2.15–2.50)
PTH (ng/l)	94.8 (37.4)	(15.0–57.0)
Albumin (g/l)	44.9 (6.7)	(35.0–52.0)
Phosphorus (mmol/l)	0.8 (0.2)	(0.81–1.45)
Vitamin D (μg/l)	27.0 (11.6)	(>30)
Creatinine (μmol/l)	81.0 (27.0)	(45.0–84.0)
Creatinine clearance (ml/min/1.73m^2^)	71.1 (18.8)	(>90)
Cervical ultrasound result		
Negative, n (%)	16 (64.0)	
Inconclusive, n (%)	9 (36.0)	
Concomitant thyroid nodules, n (%)	12 (48.0)	
MIBI SPECT/CT result		
Negative, n (%)	22 (88.0)	
Inconclusive, n (%)	3 (12.0)	

*BMI* body mass index, *PTH* parathyroid hormone

### FCH PET/CT

For the FCH PET/CT acquisition, the injected FCH activity was 113 ± 33 MBq, and the delay between injection and acquisition was 63 ± 8 min. Effective doses due to the FCH injection and the low-dose CT were 2.67 ± 2.50 mSv and 1.14 ± 0.35 mSv, respectively.

**Fig. 1:**
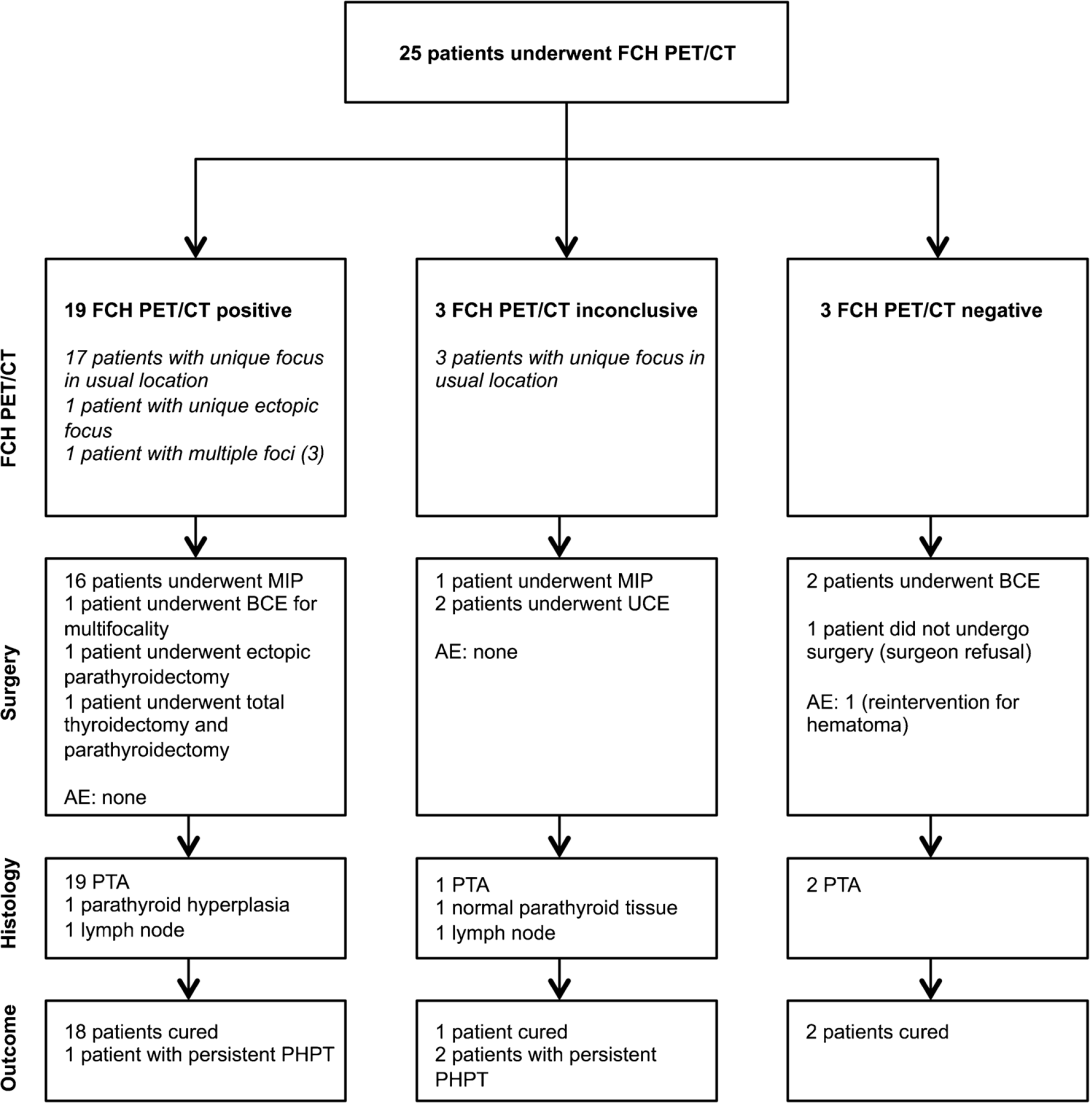
Flow chart of patients through the study. *MIP* minimally invasive parathyroidectomy, *BCE* bilateral cervical exploration, *UCE* unilateral cervical exploration, *AE* adverse event, *PTA* parathyroid adenoma, *PHPT* primary hyperparathyroidism

FCH PET/CT showed 21 positive foci and 3 inconclusive foci in 22 patients, with a maximum transverse CT diameter of 13.1 ± 8.6 mm. The FCH PET/CT results and the flow of patients through the study are depicted in Fig. 1. The inter observer agreement was considered almost perfect when the inconclusive results were classified either as positive (κ = 0.92 (95%CI: 0.73–0.99)) or negative (κ = 0.88 (95%CI: 0.68–0.97)), and considered substantial when all three groups were taken into account (κ = 0.79 (95%CI: 0.63–0.90)).

**Fig. 2:**
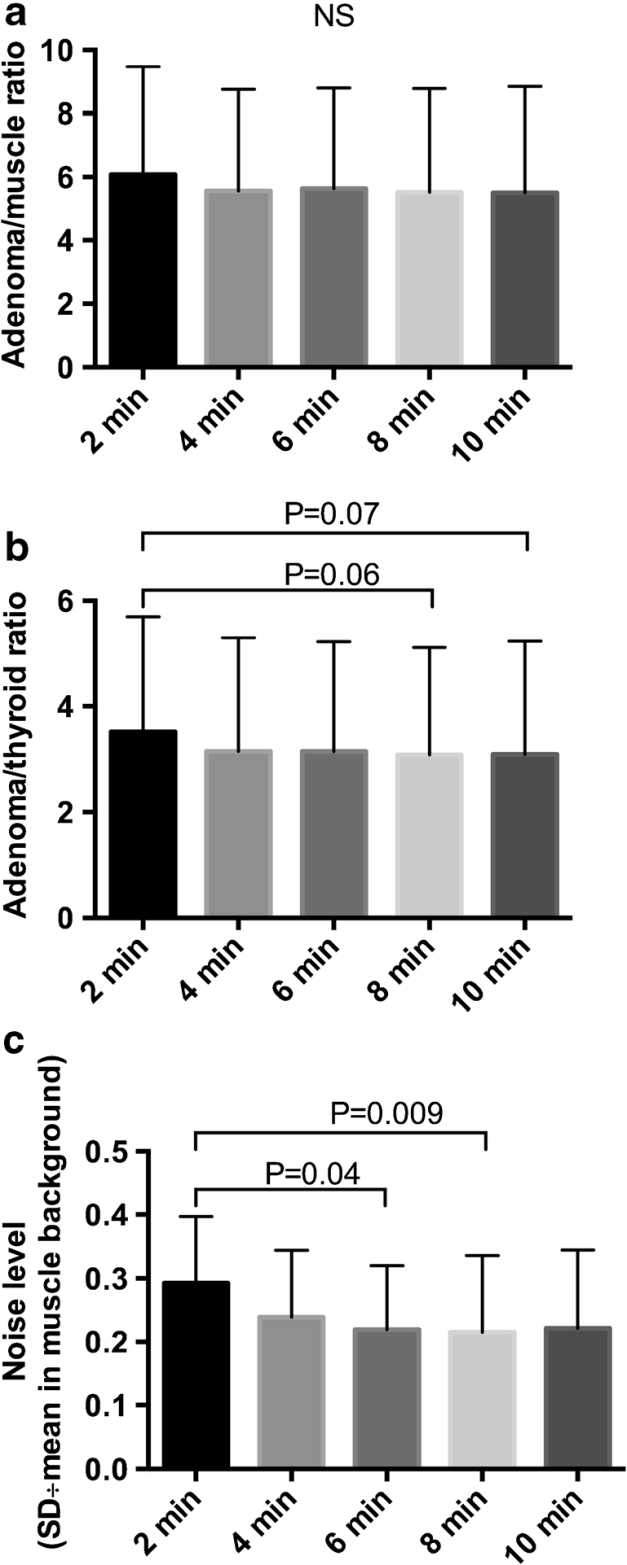
Semi-quantitative analyses of the (**a**) PTA-to-muscle ratio (**b**) PTA-to-thyroid ratio and (**c**) noise level in the muscle background for the list-mode PET acquisitions reconstructed with an increment of 2 min

SUV_max_ of PTAs on the 10 min reconstruction was 6.73 ± 3.27. SUV_mean_ in the thyroid background and in surrounding muscle background were 2.43 ± 0.63 and 1.36 ± 0.49, respectively. No statistical significance was observed between PTA/muscle ratios for the different reconstructions. With regards to the PTA/thyroid ratio, a trend towards higher ratio for the 2 min reconstruction (3.51 ± 2.18) versus 8 min (3.08 ± 2.03) and 10 min (3.09 ± 2.15) reconstructions was observed, but statistical significance was not reached. The results of the semi-quantitative analysis of the list-mode PET data are shown in Fig. 2.

### Surgery

Surgery was performed 50 ± 49 days after the FCH PET/CT. One patient did not undergo surgery. The FCH PET/CT result guided surgery in 22 patients (88%). The different surgical procedures and the adverse events can be found in Fig. 1. The mean duration of the surgical procedure was 44 ± 24 min for the MIP and 100 ± 45 min for the other surgical procedures (*p* = 0.0096).

### Histology

The results of the histopathological analysis of the resected specimens can be found in Fig. 1.

### Diagnostic performance

When dichotomizing the FCH PET/CT results, thereby classifying the inconclusive FCH PET/CT results as positive, 21 foci were considered true positive (TP), 0 true negative (TN), 3 false positive (FP) and 2 false negative (FN), resulting in per lesion and per patient sensitivities of 91.3% (95%CI: 72.0–98.9) and 90.5% (95%CI: 69.6–98.8), respectively, of FCH PET/CT for PTA detection. The corresponding per lesion and per patient positive predictive values were 87.5% (95%CI: 67.6–97.3) and 86.4% (95%CI: 65.1–97.1), respectively.

In the FCH PET/CT positive group, the FP result occurred in a patient with a previous history of thyroidectomy. The FCH-PET/CT showed a single PTA in the right superior position that was not found at surgery. PHPT and a right superior FCH positive focus persisted after surgery.

The FN results occurred in two patients with negative FCH PET/CT and a 15 mm left superior and 16 mm right superior PTA at BCE.

Three patients had an inconclusive FCH PET/CT result, two with faint left inferior uptake, corresponding to one normal parathyroid and one PTA, and one with faint right inferior uptake corresponding to a lymph node.

All site classifications were concordant, except for one patient in whom the FCH PET/CT position was classified as right inferior, whereas the surgical classification was right superior.

Four patients had a previous history of thyroid or parathyroid surgery. One of the two patients with previous thyroid surgery corresponded to the patient with the FP result described above, and the other patient had a previous history of a right hemithyroidectomy for vesicular adenoma. FCH PET/CT showed a TP right inferior PTA, which was removed by MIP, leading to cure. One of the two patients with previous parathyroid surgery was a patient with familial hyperparathyroidism (HRPT2 gene mutation) and a previous upper left PTA resected by MIP, with persistence of PHPT after surgery. FCH PET/CT showed a TP left inferior PTA of 9 mm, which was resected by MIP leading to cure. The other patient had a previous history of parathyroidectomy by BCE. Conventional imaging and FCH PET/CT results were all negative, and surgery was not performed.

### Outcome

**Fig. 3:**
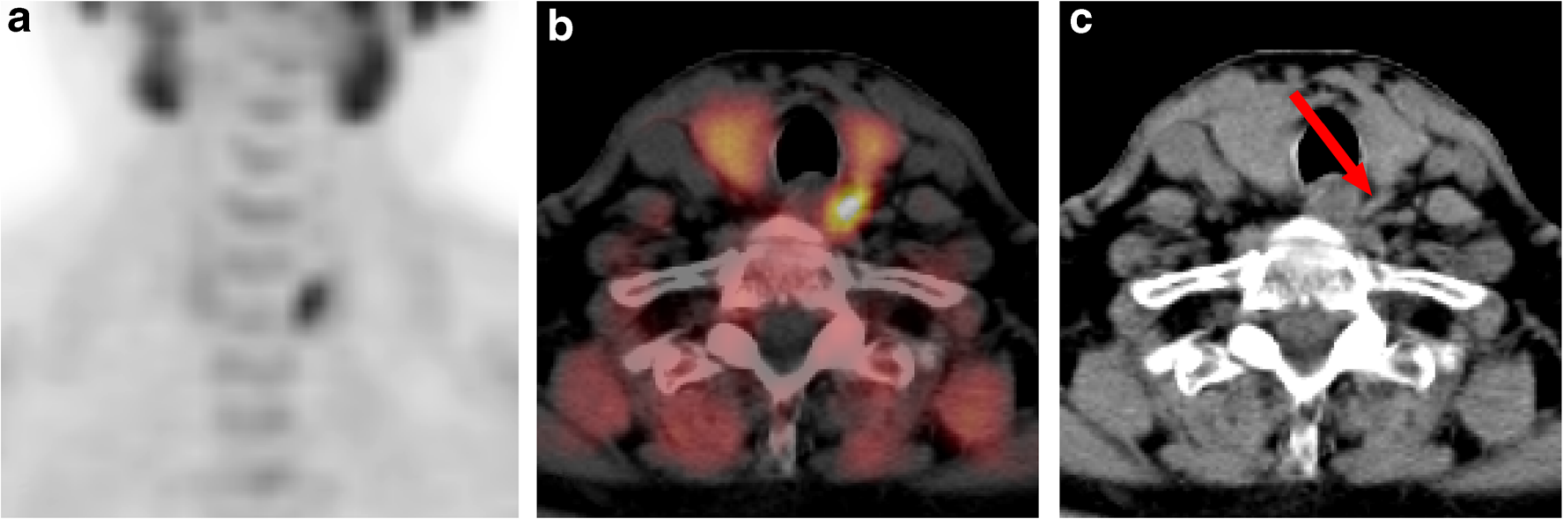
FCH PET/CT images of a 66-y-old male patient with PHPT and negative cervical ultrasound and MIBI SPECT/CT. (**a**) FCH PET maximum-intensity-projection, (**b**) FCH PET/CT fusion transverse slice, and (**c**) low-dose CT transverse slice, showing increased FCH uptake in a flat PTA in the left superior position (red arrow). Resection of a flat left superior PTA was performed by minimally invasive parathyroidectomy, leading to cure

**Fig. 4:**
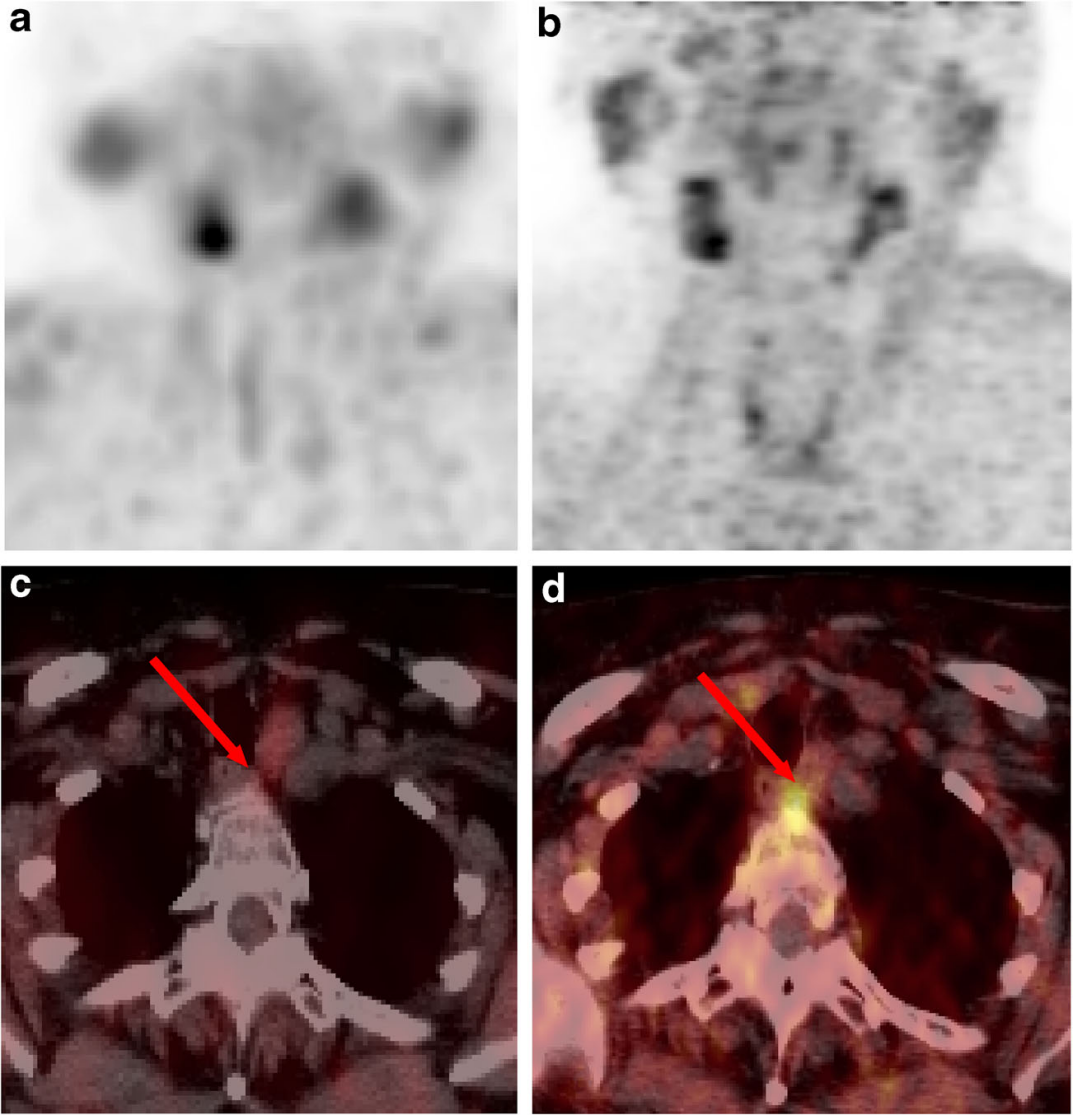
MIBI SPECT/CT and FCH PET/CT images of a 68-y-old female patient with PHPT, an inconclusive cervical ultrasound report (dubious right inferior focus only visible on one plane) and a negative MIBI SPECT/CT. (**a**) MIBI SPECT maximum-intensity-projection, (**c**) MIBI SPECT/CT transverse slice, (**b**) FCH PET maximum-intensity-projection, and (**d**) FCH PET/CT transverse slice. The MIBI and FCH MIP images (**a**, **b**) did not show clear focal uptake in the lower neck. However, the FCH PET/CT transverse slice (**d**) showed increased FCH uptake in a small ectopic deep-laying left-sided PTA in the upper mediastinum, which was negative on MIBI SPECT/CT (**c**) (red arrows). Successful ectopic parathyroidectomy was performed, leading to cure

Overall, 21 (88%) of the patients were considered cured after surgery. Two cases are illustrated in Figs. 3 and 4. The serum calcium level after surgery in the patients considered cured was 2.36 ± 0.17 mmol/l, versus 2.72 ± 0.06 mmol/l in the patients with persistent PHPT. BCE could be avoided in 18 (75%) patients (17 MIPs and 1 ectopic parathyroidectomy).

## Discussion

Our study shows that FCH PET/CT can successfully guide parathyroidectomy in patients with PHPT and negative or inconclusive cervical ultrasound and MIBI SPECT/CT results, due to its high detection rate, sensitivity and PPV of FCH-PET/CT for PTA localization. In 75% of patients, a BCE could be avoided.

For this study, a homogeneous group of biologically proven PHPT patients was prospectively recruited. All patients underwent the same MIBI SPECT/CT and FCH PET/CT imaging protocol, and all but one underwent surgery. Most patients could benefit from an outpatient MIP, a surgical procedure that was about two times less time-consuming than the other surgical procedures, and for which no adverse events were reported. This procedure almost always led to cure. The only patient with a positive FCH PET/CT without cure after surgery was a patient with a persistent right superior FCH positive focus. Because of lacking histological proof for PTA, the FCH PET for this patient was classified as FP, although we might have rather classified the surgery as FN. For the three patients with inconclusive FCH PET/CT results, one patient underwent a successful MIP, whereas the two other patients underwent unsuccessful unilateral cervical exploration (UCE). Caution should thus be taken with inconclusive FCH PET/CT results, and the inconclusive result should lead to a BCE starting on the site of the dubious focus, rather than to MIP or UCE. For the three patients with negative FCH PET/CT results, two patients underwent successful BCE, and one patient continued active surveillance (the surgeon refused to reintervene because of negative imaging and a previous history of parathyroidectomy by BCE).

FCH PET images of adenomas displayed high contrast: on average, FCH uptake in adenomas was 5.5 fold higher than surrounding muscle background and 3 fold higher than thyroid uptake, making interpretation of PET images confident in most cases; only 3 (12%) doubtful cases were reported. A secondary objective of this study was to define the optimal acquisition time with a fixed low dose of 1.5 MBq/kg of FCH. Using PSF modeling that is available from all major PET vendors [20], we did not find an improvement in the PTA-to-background ratio for a long acquisition time compared to a standard acquisition time. Thus, a standard acquisition time seems feasible. However, it should be noted that a 2mm^3^ voxel size was used and is likely to have improved the detection of adenomas. Of note, the radiation dose delivered to the patient was very low (total effective dose: 3.81 ± 2.42 mSv).

Our study has several limitations. Firstly, the number of eligible patients was not recorded. The APACH1 protocol was only proposed to patients with PHPT and negative or inconclusive conventional imaging results with an indication for surgical management. Secondly, the delay of four weeks between the FCH PET/CT and surgery could not always be respected, for practical reasons and the absence of medical urgency. However, we feel this protocol violation did not significantly influence the results. Thirdly, in theory, for more precise estimates of the diagnostic performance of FCH PET/CT for PTA localization, BCE should have been performed in all patients. For ethical reasons though, this did not seem feasible. Furthermore, intra-operative PTH measurements were not available in both centers. Lastly, long term follow-up to confirm the maintenance of normocalcemia was not part of our prospective study protocol.

To our knowledge, this is the largest prospective study showing the value of FCH PET/CT as a second line tracer for PTA detection in PHPT. Other, mostly retrospective, studies not limited to PHPT show equally promising results [11, 12, 21, 22]. In the retrospective study by Kluijfhout et al. on 44 patients with hyperparathyroidism and inconclusive ultrasound and MIBI scintigraphy, FCH PET/CT was positive in 77% of cases, correctly localizing 33 of 35 abnormal glands with a PPV of 97% [21]. Michaud et al. found a per lesion sensitivity of FCH PET/CT for PTA or hyperplasia detection of 89% in 12 patients with primary or secondary hyperparathyroidism, with a detection rate on the per patient level of 92% [11].

A head-to-head comparison of FCH PET/CT and parathyroid scintigraphy was performed by Lezaic et al. [12] in 24 patients with PHPT, showing a largely superior sensitivity of FCH-PET/CT versus scintigraphy for PTA or hyperplasia detection (92% for FCH PET/CT versus 64% for a combined read of three frequently used scintigraphy protocols). FCH PET/CT was found to be especially valuable in the detection of multiglandular disease, a frequent finding in their study population (29%) compared to ours (4%). The TP identification of multisite disease by FCH PET/CT might avoid failure of surgery. In our study population, failure of surgery could be avoided in a patient with multiple bilateral PTAs, as well as in a patient with an ectopic PTA in the upper mediastinum (Figure 4).

Another PET tracer studied for preoperative PTA detection is C11-methionine. Reported sensitivities from two recent meta-analyses vary from 69% to 81%, and the per-patient detection rate was estimated at 70% [23, 24]. Thus, it seems that FCH PET/CT has a superior performance than C11-methionine PET/CT for PTA detection. In terms of availability, the balance is largely in favor of the widely available tracer FCH, as C11-methionine production requires an on-site cyclotron due to its short half-life.

The question whether FCH PET/CT could replace MIBI SPECT/CT as a first line imaging modality in PHPT should be the subject of future prospective studies, ideally including an analysis of the associated health care costs. Although our data suggest the superior diagnostic performance of FCH PET/CT compared to ultrasound and MIBI SPECT/CT for PTA detection, its costs are largely superior too, and the gain in avoided BCEs or complications due to persistent PHPT should outweigh the additional expenses.

## Conclusion

Preoperative FCH PET/CT has a high sensitivity and PPV for PTA detection in patients with PHPT and negative or inconclusive cervical ultrasound and MIBI SPECT/CT results. For PTA imaging, a FCH PET/CT protocol with a low injected FCH dose and a standard acquisition time seems feasible. Bilateral cervical exploration could be avoided in the majority (75%) of patients.
